# Evaluation of dietary intake assessed by the Dutch self-administered web-based dietary 24-h recall tool (Compl-eat™) against interviewer-administered telephone-based 24-h recalls

**DOI:** 10.1017/jns.2017.45

**Published:** 2017-09-19

**Authors:** Saskia Meijboom, Martinette T. van Houts-Streppel, Corine Perenboom, Els Siebelink, Anne M. van de Wiel, Anouk Geelen, Edith J. M. Feskens, Jeanne H. M. de Vries

**Affiliations:** Division of Human Nutrition, Wageningen University, PO Box 17, 6700 AA Wageningen, The Netherlands

**Keywords:** 24-h Dietary recalls, Comparative studies, Dietary assessment, Internet, 24 hR, 24-h dietary recall, EI, energy intake, ICC, intra-class correlation, LCC, Lin's concordance correlation coefficient, NQplus, Nutrition Questionnaires plus

## Abstract

Self-administered web-based 24-h dietary recalls (24 hR) may save a lot of time and money as compared with interviewer-administered telephone-based 24 hR interviews and may therefore be useful in large-scale studies. Within the Nutrition Questionnaires plus (NQplus) study, the web-based 24 hR tool Compl-eat™ was developed to assess Dutch participants’ dietary intake. The aim of the present study was to evaluate the performance of this tool against the interviewer-administered telephone-based 24 hR method. A subgroup of participants of the NQplus study (20–70 years, *n* 514) completed three self-administered web-based 24 hR and three telephone 24 hR interviews administered by a dietitian over a 1-year period. Compl-eat™ as well as the dietitians guided the participants to report all foods consumed the previous day. Compl-eat™ on average underestimated the intake of energy by 8 %, of macronutrients by 10 % and of micronutrients by 13 % as compared with telephone recalls. The agreement between both methods, estimated using Lin's concordance coefficients (LCC), ranged from 0·15 for vitamin B_1_ to 0·70 for alcohol intake (mean LCC 0·38). The lower estimations by Compl-eat™ can be explained by a lower number of total reported foods and lower estimated intakes of the food groups, fats, oils and savoury sauces, sugar and confectionery, dairy and cheese. The performance of the tool may be improved by, for example, adding an option to automatically select frequently used foods and including more recall cues. We conclude that Compl-eat™ may be a useful tool in large-scale Dutch studies after suggested improvements have been implemented and evaluated.

In nutritional epidemiological studies investigating the relationship between diet and disease, the collection of high-quality dietary intake data is important. A FFQ is often used in these large-scale studies because it is a cost-effective method for self-reported dietary intake^(^^1^^)^. However, as suggested by biomarker-based validation studies, the 24-h dietary recall (24 hR) method shows less under-reporting of dietary intake than FFQ^(^^2^^–^^4^^)^.

The 24 hR is open-ended and provides detailed information on types of food intake and amounts consumed during the past 24 h. Repeating 24 hR in one individual enables the estimation of usual intake because day-to-day variation can be taken into account^(^^5^^)^. However, the 24 hR administered by telephone or face to face is a relatively expensive method because of the workload and the costs incurred in employing trained dietitians to conduct the interviews and code the foods^(^^6^^)^.

The use of technology is considered an important step forward in the assessment of dietary intake. Web-based 24 hR allow self-administered dietary assessment at a time and a location that are convenient for participants. To fill out a web-based recall, participants only require access to the Internet. As the costs of using the method are also lower because interviews and automated coding of the consumption data are not required, web-based 24 hR are feasible for large-scale dietary studies^(^^1^^)^.

In the last decade, several web-based self-administered 24 hR tools have been developed in different countries to assess dietary intake in adults^(^^7^^)^. Examples are ASA24, myfood24, DietDay, Nutrinet-Santé, Oxford WebQ and IMM^(^^8^^–^^13^^)^. These tools, except Oxford WebQ, are modelled on the multiple-pass method^(^^14^^)^. In this method, participants first fill in a quick list of foods consumed and in a following step describe the type and amount of these foods in more detail.

The Nutrition Questionnaires plus (NQplus) study is a longitudinal observational study performed within the surroundings of Wageningen, the Netherlands. One of the aims of the NQplus study is to develop a national dietary assessment reference database for future development and improvement of dietary assessment methods^(^^15^^)^. In this study, a web-based self-administered 24 hR tool called Compl-eat™ was developed to assess dietary intake of participants with a Dutch food pattern. Compl-eat™ was also modelled on the multiple-pass method.

The objective of the present study was to evaluate the ability of the first version of this self-administered web-based 24 hR Compl-eat™ to assess the intake of foods and nutrients in the NQplus study. We used the interviewer-administered telephone-based 24 hR as the reference method.

## Methods

### Participants

The NQplus study was conducted between May 2011 and June 2015. Participant recruitment was spread over more than 2 years. A total of 2048 men and women aged between 20 and 70 years and able to speak and write Dutch were included^(^^15^^)^. Half of them (*n* 1089) were randomly allocated to the recall group. In the recall group, participants were asked to complete multiple 24 hR. For the present study, we used the intake data of all participants from this recall group who completed three web-based and three telephone-based 24 hR by 1 March 2014. The characteristics of this selection of 514 participants are similar to those of the complete NQplus population. The ethical committee of Wageningen University approved the study. All participants gave written informed consent.

### Study design

The days for collecting the 24 hR were randomly selected and scheduled across the year. Distribution of the collection days across spring, summer, autumn and winter was 30, 32, 22 and 16 %, respectively, for the web-based 24 hR, and 16, 32, 25 and 27 % for the telephone-based 24 hR. The recall days for both methods were randomly allocated to the participants. Therefore, the sequence in which the web-based and the telephone-based 24 hR were conducted as well as the number of days between the recalls varied per participant. The average number of days between the first and the last recall was 354 d. Scheduling recall days over weekdays and weekend days was not taken into account but appeared to be distributed rather evenly (70 % on weekdays and 30 % on weekend days) for both web-based and telephone-based 24 hR.

We compared the intakes of energy, nutrients and food groups for the three web-based 24 hR with the intakes for the three telephone-based 24 hR.

### Methods of dietary assessment

In both the web-based and the telephone-based 24 hR, the multiple-pass approach was used; this is a validated technique to increase the accuracy of recalls^(^^14^^)^. In both recall methods, portion sizes were reported in commonly used household measures, standard portions, weight in g or volume in litres^(^^16^^)^.

#### Web-based 24-h recalls

The default language of the web-based 24 hR module Compl-eat™ is Dutch. NQplus study participants were invited unannounced via an email, sent at 06.00 hours, to report their previous day's intake from waking up until waking up the next morning. The questionnaire was accessible until midnight the same day. Before the participants started to report their intake, they could view two instruction videos. The first instruction video (2 min 16 s) explained how the participants should fill in the quick list. The second video (2 min 26 s) showed how to fill in the details (type and amount) of the foods consumed. The Compl-eat™ 24 hR module guided participants to report all foods and drinks consumed during the previous day. The tool allowed participants to select foods and standard recipes commonly used by the Dutch population^(^^17^^)^. It contained a recipe module in which the participants could report their intake of a certain dish by choosing or adapting a standard recipe, or listing all the ingredients in their own recipe and indicating how much of the finished dish they had consumed. Yield and retention factors were automatically taken into account when appropriate. Participants could include notes for clarifications when needed. At the end of the recall, Compl-eat™ reminded the participants to fill in often forgotten foods such as sugar in coffee, snacks, fruit and cooking fat.

Trained dietitians checked all the web-based 24 hR for their completeness and unusual portion sizes and processed all notes made by the participants. The participants were not contacted for clarifications. Adjustments of errors and notes were made in a standardised way, using standard portion sizes and recipes according to a protocol. An example of an error that occurred was a report of 125 cups of coffee instead of one cup of 125 g. Notes contained, for example, a description of a food that the participant could not find on the food list.

#### Telephone-based 24-h recalls

The telephone-based 24 hR in the NQplus study were conducted unannounced by trained dietitians. After a maximum of ten recall attempts at different times and dates, a telephone appointment was made for the interview. This happened in less than 1 % of the telephone-based 24 hR. The recalls were transcribed into food codes and amounts^(^^16^^,^^18^^)^. Regular meetings with all dietitians ensured the quality of the interviews and the food coding. All dietitians coded the same 24 hR, and differences in coding were discussed during these meetings. At least one interview per dietitian was tape recorded with the participant's permission and reviewed for quality by a senior research dietitian.

#### Computation of the intake data

The data from both the self-administered web-based and the interviewer-administered telephone-based 24 hR in the NQplus study were entered and calculated in the Compl-eat™ computation module. This module consists of a data-entry part for researchers and a food calculation system and is able to generate output for different purposes.

Foods were aggregated into food groups according to the Dutch food composition database.

In addition, total energy and nutrient intakes from both the web-based and the telephone-based 24 hR were calculated in Compl-eat™ by multiplying intakes by nutrient composition using the same Dutch food composition database (NEVO-database, 2011)^(^^18^^)^.

For both the web-based and the telephone-based 24 hR, a data check was performed to identify outliers. Recalls with the highest and the lowest energy and nutrient intakes were identified to evaluate whether the reported foods and amounts were within normal ranges of intake. Recalls with intakes outside the normal ranges were examined in detail to find implausible amounts of a certain food (e.g. 150 ml of syrup instead of 150 ml of lemonade made with syrup and water). During this check, additional information on dietary regimens and special occasions such as birthdays or holidays was taken into consideration. About 0·5 % of the web-based 24 hR were considered incomplete, for example if the participants had stopped filling in the recall or because of a low reported intake due to illness on the recall day. These incomplete web-based recalls were removed from the analysis. For the telephone-based 24 hR, no incomplete recalls occurred. The interview was postponed if the participant had been ill the previous day, and no participant terminated the recall before the interview was finished.

### Demographic and anthropometric variables

Information on education level (low: primary school, vocational or lower general secondary education; moderate: higher secondary education or intermediate vocational training; high: higher vocational education or university), smoking habits (never, current, former) and medical history was collected using self-administered questionnaires. Furthermore, participants came to the study centre to be measured for height and weight. Trained research assistants performed all measurements. Height was measured to the nearest 0·1 cm without shoes using a stadiometer (SECA). Body weight was measured without shoes and sweaters and with empty pockets to the nearest 0·1 kg on a digital scale (SECA). BMI (kg/m^2^) was calculated and three categories were defined: BMI <25 kg/m^2^ (normal weight), BMI 25–30 kg/m^2^ (overweight) and BMI ≥30 kg/m^2^ (obese).

### Statistical analysis

Mean crude and energy-adjusted dietary intakes and standard deviations were calculated for the average of the three web-based and the three telephone-based 24 hR; the residual method was used to estimate energy-adjusted intakes^(^^19^^)^.

To evaluate the self-administered web-based against the interviewer-administered telephone-based 24 hR, several analyses were carried out. First, absolute differences in mean dietary intake between the web-based and telephone-based 24 hR were tested by paired *t* tests. Second, the agreement between the two recall methods was visualised by plotting the difference against the mean of the two methods in Bland–Altman plots^(^^20^^)^. Third, using Lin's concordance correlation coefficients (LCC), the agreement between the results of the two methods was evaluated by measuring the variation from the 45° line through the origin for energy and nutrient intake as well as for intake in absolute amounts of food groups^(^^21^^)^. The associations determined with LCC were judged as fair if the correlation coefficients were 0·4–0·7 and good if they were at least 0·7^(^^13^^,^^19^^)^. Mean LCC for macronutrients, micronutrients and food groups were calculated by averaging LCC for eighteen macronutrients, thirteen micronutrients and twenty-two food groups, respectively^(^^22^^)^. Fourth, the between-subject variability and the within-subject variability in dietary intake were estimated for each recall method, and intra-class correlations (ICC), including 95 % CI, were calculated. Fifth, regression analysis was used to determine the relationships of sex, age, BMI category, occurrence of chronic diseases and education level to the difference in energy intake (EI) between the web-based and the telephone-based 24 hR.

To further evaluate under-reporting for each participant, the EI:BMR ratio and a cut-off value for EI:BMR ratio were calculated. For this, we took into account the physical activity level (PAL, expressed as multiples of BMR), the between-subject variation in PAL and the within-subject variation in EI. We assumed a within-subject variation in estimated BMR of 8·5 %^(^^23^^)^. Henry's^(^^24^^)^ standard equation was used to predict the participants’ BMR. A mean EI:BMR ratio of a (sub)population below the cut-off value was interpreted as under-reporting of EI.

All analyses were carried out using IBM SPSS Statistics, version 21 (IBM Corporation).

## Results

The average age of participants was 54 (sd 11) years and ranged from 21 to 72 years. Almost half of them were men, and half of the participants were overweight or obese. Two-thirds of the participants had a high education level (Table 1).

**Table 1.** Characteristics of Nutrition Questionnaires plus (NQplus) study participants providing recall data (Numbers of participants and percentages; mean values and standard deviations)

	Participants with three web-based and three telephone-based 24-h recalls					
	All		Men		Women		
	*n*	%	*n*	%	*n*	%
Participants	514	100	238	46	276	54
Age (years) (*n* 514)						
Mean	54		58		51	
sd	11		10		12	
BMI (kg/m^2^) (*n* 513)						
Mean	25·8		26·4		25·2	
sd	4·0		3·5		4·3	
<25 kg/m^2^	244	48	86	36	158	57
25–30 kg/m^2^	199	39	116	49	84	30
≥30 kg/m^2^	70	14	35	15	35	13
Estimated BMR (MJ/d) (*n* 513)						
Mean	6·4		7·2		5·7	
sd	1·0		0·8		0·6	
PAL (multiple of BMR) (*n* 513)						
Mean	1·64		1·62		1·66	
sd	0·19		0·19		0·19	
Cigarette smoking (*n* 464)						
Never	232	50	85	39	147	60
Former	193	42	108	50	85	34
Current	39	8	24	11	15	6
Presence of chronic diseases (*n* 508)						
Cancer	26	5	9	4	17	6
Diabetes mellitus	18	4	11	5	7	3
Heart attack	13	3	10	4	3	1
Education level (*n* 511)						
Low	23	5	13	6	10	4
Intermediate	140	27	58	24	82	30
High	348	68	167	70	181	66

PAL, physical activity level.

### Comparison of the assessment of energy and nutrients between methods

**Fig. 1.**
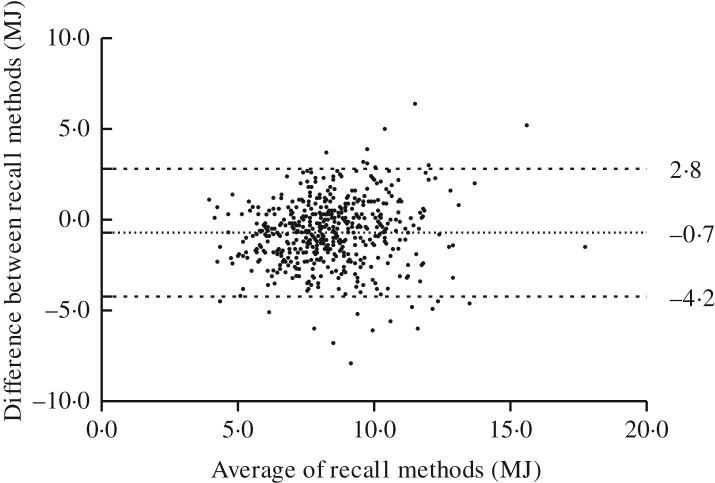
Bland–Altman plot of total energy intake estimated with the self-administered web-based and interviewer-administered telephone-based 24-h dietary recalls.

The EI estimated with the web-based 24 hR was on average 8·2 % lower than that assessed by the telephone-based recalls (Table 2). The Bland–Altman plot, presenting the average EI according to the web-based and the telephone-based recall plotted against the difference of the two methods for total EI (Fig. 1), showed 95 % limits of agreement of −4·2 and 2·8 MJ. The difference in mean EI increased slightly with increasing mean EI in both methods (slope 0·107; *P* = 0·014).

**Table 2.** Differences in total energy and nutrient intakes and Lin's concordance correlation coefficients (LCC) between the self-administered web-based 24-h dietary recall and the interviewer-administered telephone-based 24-h dietary recalls (*n* 514) (Mean values and standard deviations, proportional differences and LCC)

	Web-based 24-h recalls*		Telephone-based 24-h recalls*		Difference between both recall methods*				LCC*
	Mean	sd	Mean	sd	Mean	sd	%†	*P*	
Energy									
kcal	1911	507	2081	465	−171	430	−8·20	<0·0001	0·57
kJ	8014	2122	8728	1947	−714	1795	−8·18	<0·0001	0·58
Macronutrients									
Total protein (g)	74·8	11·8	82·2	13·3	−7·46	13·6	−9·07	<0·0001	0·35
Vegetable protein (g)	32·1	6·95	33·8	6·89	−1·64	6·58	−4·86	<0·0001	0·53
Animal protein (g)	42·8	13·7	48·4	14·9	−5·69	15·2	−11·7	<0·0001	0·41
Total fat (g)	70·3	11·7	79·4	11·7	−9·16	12·5	−11·5	<0·0001	0·33
SFA (g)	27·0	6·10	29·3	5·88	−2·29	6·01	−7·83	<0·0001	0·46
MUFA (g)	23·8	5·79	27·2	5·73	−3·38	6·38	−12·4	<0·0001	0·33
PUFA (g)	12·9	3·87	15·8	4·25	−2·89	4·71	−18·3	<0·0001	0·26
Linoleic acid (g)	10·5	3·37	12·8	3·77	−2·35	4·13	−18·3	<0·0001	0·27
*Trans*-fatty acids (g)	1·36	0·73	1·30	0·63	0·07	0·85	5·30	0·07	0·22
α-Linolenic acid (g)	1·36	0·48	1·75	0·56	−0·39	0·62	−22·4	<0·0001	0·23
EPA (g)	0·10	0·21	0·12	0·19	−0·02	0·23	−17·2	0·05	0·30
DHA (g)	0·13	0·33	0·17	0·30	−0·04	0·38	−22·3	0·02	0·26
Cholesterol (g)	203	102	212	95·6	−9·29	115	−4·37	0·07	0·32
Total carbohydrates (g)	210	32·2	223	33·7	−13·5	30·5	−6·05	<0·0001	0·53
Mono- and disaccharides (g)	86·9	25·6	100	27·3	−13·1	24·9	−13·1	<0·0001	0·50
Polysaccharides (g)	123	24·8	123	22·6	−0·42	23·3	−0·34	0·68	0·52
Dietary fibre (g)	21·6	5·68	23·2	5·83	−1·59	5·14	−6·85	<0·0001	0·58
Alcohol (g)	12·6	14·7	12·7	13·5	−0·05	10·9	−0·39	0·92	0·70
Micronutrients									
Ca (mg)	926	266	1048	286	−122	258	−11·6	<0·0001	0·52
K (mg)	3131	579	3407	570	−275	575	−8·08	<0·0001	0·45
β-Carotene (μg)	2263	2703	2328	2484	−65·6	3100	−2·82	0·63	0·29
Vitamin B_1_ (mg)	0·98	0·36	0·99	0·28	−0·01	0·42	−1·15	0·54	0·15
Vitamin B_2_ (mg)	1·37	0·37	1·49	0·40	−0·13	0·40	−8·51	<0·0001	0·45
Vitamin B_6_ (mg)	1·56	0·54	1·73	0·54	−0·17	0·58	−9·71	<0·0001	0·40
Vitamin B_12_ (μg)	4·25	3·00	5·07	3·64	−0·82	3·94	−16·1	<0·0001	0·30
Vitamin D (μg)	3·08	1·86	3·36	1·88	−0·28	2·27	−8·38	0·01	0·26
Vitamin E (mg)	11·0	4·20	13·1	4·18	−2·01	4·88	−15·4	<0·0001	0·29
Vitamin C (mg)	89·5	49·8	98·3	49·2	−8·76	54·3	−8·91	0·00	0·39
Lycopene (μg)	1342	2106	2165	3317	−823	3429	−38·0	<0·0001	0·23
Retinol activity equivalents (μg)	730	617	800	595	−70·4	809	−8·79	0·05	0·11
Folic acid equivalents (μg)	254	86·0	298	97·2	−44·7	97·4	−15·0	<0·0001	0·39

* Intakes of nutrients and LCC are based on energy-adjusted values; energy-adjusted intakes were calculated using the residual method.

† Proportional difference is calculated as ((mean of web-based 24 h dietary recall) – (mean of telephone-based 24 h dietary recall))/(mean of telephone-based 24 h dietary recall) × 100.

For all nutrients, except for *trans*-fatty acids, the web-based 24 hR estimated lower intakes than the telephone-based recall, and most differences were statistically significant (Table 2). The average difference in macronutrients was −10 % and varied from −22·4 % for α-linolenic acid to −0·34 % for polysaccharides. For micronutrients, the average difference was −12 % and varied from −38 % for lycopene to −1·15 % for vitamin B_1_.

To investigate how well participants were ranked according to their intake, LCC was calculated between the self-administered web-based and the interviewer-administered telephone-based 24 hR, and was 0·58 (95 % CI 0·52, 0·63) for EI (Table 2). For the nutrients it was lower: on average 0·39 and 0·46 for the macronutrients, and 0·33 and 0·36 for the micronutrients based on energy-adjusted and not energy-adjusted values, respectively. For macronutrients, the energy-adjusted LCC ranged from 0·22 for *trans*-fatty acids intake to 0·70 for alcohol intake; for micronutrients, energy-adjusted LCC ranged from 0·11 for retinol activity equivalent intake to 0·52 for Ca intake.

ICC provide information on within-person variation within a recall method. In the 3-d web-based 24 hR, the ICC for energy was 0·41. For macronutrients, the ICC ranged from 0·05 for EPA and DHA intake to 0·57 for alcohol intake, and for micronutrients from 0·07 for β-carotene to 0·39 for K intake. For the telephone-based 24 hR, rather similar ICC to those of the web-based recalls were found (Table 3).

**Table 3.** Intra-class correlations (ICC) of nutrient intakes for the three self-administered web-based and the three interviewer-administered telephone-based 24-h dietary recalls (*n* 514) (Correlations and 95 % confidence intervals)

	Web-based 24-h recalls		Telephone-based 24-h recalls	
	ICC*	95 % CI	ICC*	95 % CI
Energy				
kcal	0·41	0·35, 0·46	0·40	0·34, 0·45
kJ	0·41	0·36, 0·46	0·40	0·34, 0·45
Macronutrients (g)				
Total protein	0·32	0·26, 0·38	0·31	0·26, 0·37
Vegetable protein	0·41	0·36, 0·46	0·44	0·39, 0·49
Animal protein	0·24	0·19, 0·30	0·26	0·20, 0·32
Total fat	0·29	0·24, 0·35	0·26	0·21, 0·32
SFA	0·32	0·27, 0·38	0·26	0·20, 0·32
MUFA	0·25	0·20, 0·31	0·22	0·16, 0·28
PUFA	0·22	0·16, 0·28	0·24	0·18, 0·30
Linoleic acid	0·21	0·15, 0·26	0·22	0·17, 0·28
*Trans*-fatty acids	0·15	0·10, 0·21	0·07	0·02, 0·13
α-Linolenic acid	0·20	0·15, 0·26	0·23	0·17, 0·29
EPA	0·05	0·00, 0·10	0·05	0·00, 0·10
DHA	0·05	0·00, 0·10	0·04	−0·010·09
Cholesterol	0·16	0·11, 0·22	0·11	0·06, 0·17
Total carbohydrates	0·42	0·36, 0·47	0·47	0·42, 0·52
Mono- and disaccharides	0·40	0·35, 0·45	0·44	0·39, 0·49
Polysaccharides	0·36	0·31, 0·42	0·43	0·37, 0·48
Dietary fibre	0·39	0·34, 0·45	0·48	0·43, 0·53
Alcohol	0·57	0·52, 0·61	0·52	0·47, 0·57
Micronutrients				
Ca (mg)	0·37	0·31, 0·42	0·37	0·31, 0·42
K (mg)	0·39	0·33, 0·44	0·39	0·34, 0·45
β-Carotene (μg)	0·07	0·02, 0·13	0·14	0·09, 0·20
Vitamin B_1_ (mg)	0·16	0·10, 0·21	0·13	0·08, 0·18
Vitamin B_2_ (mg)	0·37	0·31, 0·42	0·34	0·28, 0·39
Vitamin B_6_ (mg)	0·33	0·28, 0·39	0·30	0·25, 0·36
Vitamin B_12_ (μg)	0·11	0·05, 0·16	0·09	0·04, 0·15
Vitamin D (μg)	0·12	0·07, 0·18	0·16	0·10, 0·21
Vitamin E (mg)	0·24	0·18, 0·29	0·20	0·14, 0·26
Vitamin C (mg)	0·24	0·19, 0·30	0·23	0·18, 0·29
Lycopene (μg)	0·10	0·05, 0·16	0·07	0·02, 0·12
Retinol activity equivalents (μg)	0·11	0·06, 0·17	0·06	0·01, 0·11
Folic acid equivalents (μg)	0·38	0·33, 0·43	0·41	0·35, 0·46

* ICC values are based on energy-adjusted values; energy-adjusted intakes were calculated using the residual method.

The average EI:BMR ratio for the web-based 24 hR (1·26) was lower than the same ratio for the telephone-based recalls (1·37). Both ratios were substantially below the estimated cut-off value of 1·62 for the participants in the present study. The average EI:BMR ratios were similar for participants with a low or intermediate education level and higher for those with a high education level: the ratios were 1·17, 1·18 and 1·30, respectively, for the web-based recalls and 1·35, 1·33 and 1·39, respectively, for the telephone-based recalls. The differences in EI between the web- and the telephone-based 24 hR were −15·0, −11·2 and −6·6 % for participants with a low (*n* 23), intermediate (*n* 140) and high (*n* 348) education level, respectively. For both methods, EI:BMR ratios decreased significantly (*P*<0·0001) with increasing BMI. For the BMI categories of BMI <25, 25–30 and ≥30 kg/m^2^, the EI:BMR ratios for the web-based 24-h recalls were 1·38, 1·18 and 1·04, respectively, and for the telephone-based recalls 1·50, 1·29 and 1·15, respectively. The differences in EI between the web- and telephone-based 24 hR were −7·9, −8·3 and −8·8 % for participants with BMI <25 kg/m^2^ (*n* 244), 25–30 kg/m^2^ (*n* 199) and ≥30 kg/m^2^ (*n* 70), respectively. Between males and females no significant differences were seen between the recall methods except for cholesterol intake (males 0·9, females −8·8 %; *P* = 0·04). Sex, age, BMI category and occurrence of chronic diseases did not contribute significantly to the variance in difference in EI between the methods. However, for education level, we found a significant contribution (*R*^2^ 0·013; *P* = 0·009).

### Comparison of the assessment of food intake between methods

The average number of reported foods per recall was lower in the web-based 24 hR (twenty foods, range 8–32) than in the telephone-based 24 hR (twenty-three foods, range 11–46 foods).

The food group fats, oils and savoury sauces contributed most (16·4 %) to the differences in EI between the web-based and the telephone-based recalls. This percentage corresponds with a difference in daily consumption of −0·1 MJ (Table 4) or −10 g (Table 5). Large contributions to the difference in reported EI were also seen for the following food groups: sugar and confectionery (12·9 %), milk and milk products (11·1 %) and cheese (10·5 %). No notable contributions to the difference in EI (less than 1 % difference) were found for the following food groups: pastry, cake and biscuits; potatoes; composite dishes; eggs; legumes; and alcoholic beverages (Table 4). The largest absolute difference in amount eaten between both methods was found in the food group coffee, tea and water (Table 5).

**Table 4.** Difference in energy intake (kJ) of different food groups and Lin's concordance correlations (LCC) between the self-administered web-based and the interviewer-administered telephone-based 24-h dietary recalls (*n* 514) (Mean values and standard deviations, proportional differences and LCC)

	Web-based 24-h recalls		Telephone-based 24-h recalls		Difference between both recall methods				LCC
	Mean	sd	Mean	sd	Mean	sd	%*	*P*	
Alcoholic beverages	465	583	462	521	3	444	0·4	0·88	0·68
Bread	1493	669	1518	665	−25	513	−3·5	0·27	0·70
Cereal products and binding agents	433	573	471	476	−38	576	−5·3	0·14	0·40
Cheese	435	336	509	358	−74	371	−10·4	<0·0001	0·42
Coffee, tea and water	21	14	73	157	−52	155	−7·3	<0·0001	0·02
Composite dishes	253	488	254	446	0	662	−0·0	1·00	0·00
Eggs	87	128	88	129	−1	144	−0·1	0·89	0·38
Fats, oils and savoury sauces	530	361	647	351	−118	424	−16·5	<0·0001	0·28
Fish	134	248	177	278	−43	327	−6·0	0·00	0·23
Fruit	419	356	461	340	−43	321	−6·0	0·00	0·57
Fruit/vegetable juices, soft drinks	200	259	237	278	−36	250	−5·1	0·00	0·56
Legumes	35	105	30	103	5	127	0·7	0·35	0·25
Meat, meat products and poultry	661	515	725	488	−64	576	−8·9	0·01	0·34
Milk and milk products	698	436	779	465	−81	404	−11·3	<0·0001	0·59
Nuts, seeds and snacks	429	520	462	542	−32	554	−4·5	0·19	0·46
Pastry, cake and biscuits	612	530	616	501	−4	589	−0·6	0·86	0·35
Potatoes	309	333	309	322	0	418	−0·0	0·99	0·19
Savoury sandwich fillings	75	168	67	150	8	153	1·1	0·25	0·54
Soups	89	142	77	138	11	176	1·6	0·14	0·21
Soya and vegetarian products	57	151	65	160	−7	142	−1·0	0·25	0·58
Sugar and confectionery	390	365	482	420	−92	409	−12·9	<0·0001	0·45
Vegetables	162	119	189	125	−26	137	−3·7	<0·0001	0·36

* Proportional difference is calculated as ((mean of web-based 24 h recall) – (mean of telephone-based 24 h recall))/(mean difference in kJ) × 100. The mean difference between the methods is −714 kJ (Table 2).

**Table 5.** Difference in amount eaten (g/d) of different food groups between the self-administered web-based and the interviewer-administered telephone-based 24-h dietary recalls (*n* 514) (Mean values and standard deviations, proportional differences and Lin's concordance correlation coefficients (LCC))

	Web-based 24-h recalls		Telephone-based 24-h recalls		Difference between both recall methods				
	Mean	sd	Mean	sd	Mean	sd	%*	*P*	LCC
Alcoholic beverages	166	233	163	210	3	183	1·7	0·73	0·66
Bread	139	63	139	62	0	46	−0·2	0·89	0·73
Cereal products and binding agents	53	63	58	59	−4	72	−7·4	0·18	0·29
Cheese	30	23	36	25	−6	25	−15·3	<0·0001	0·42
Coffee, tea and water	1205	530	1439	587	−234	363	−16·3	<0·0001	0·73
Composite dishes	39	73	39	64	1	97	1·5	0·89	−0·01
Eggs	15	21	14	19	1	23	6·8	0·35	0·37
Fats, oils and savoury sauces	29	21	39	22	−10	27	−26·0	<0·0001	0·20
Fish	20	35	25	39	−5	46	−21·7	0·01	0·24
Fruit	156	131	168	116	−12	118	−6·9	0·03	0·54
Fruit/vegetable juices, soft drinks	127	160	152	182	−25	133	−16·6	<0·0001	0·69
Legumes	7	21	6	21	1	26	13·0	0·49	0·23
Meat, meat products and poultry	80	59	81	51	−1	62	−1·1	0·75	0·37
Milk and milk products	277	179	305	185	−28	136	−9·1	<0·0001	0·71
Nuts, seeds and snacks	22	27	23	27	−1	30	−5·0	0·38	0·36
Pastry, cake and biscuits	40	35	40	34	0	41	−0·3	0·95	0·29
Potatoes	63	59	67	60	−4	76	−5·7	0·25	0·20
Savoury sandwich fillings	3	7	3	6	1	7	18·3	0·08	0·51
Soups	65	89	53	75	12	93	23·2	<0·01	0·35
Soya and vegetarian products	15	51	17	57	−2	31	−12·3	0·13	0·84
Sugar and confectionery	23	20	29	24	−7	23	−23·1	<0·0001	0·44
Vegetables	143	95	161	100	−18	104	−11·1	<0·001	0·42

* Proportional difference is calculated as ((mean of web-based 24 h recall) – (mean of telephone-based 24 h recall))/(mean of telephone-based 24 h recall) × 100.

The LCC for the contribution to EI and for the amount consumed by the food group composite dishes between the two recall methods was lower than that for the other food groups (Tables 4 and 5). The largest LCC was found for the food group bread (*r* 0·70) with respect to contribution to EI, and for the food group soya foods and meat substitutes with respect to the amount consumed (*r* 0·84). The average LCC between the methods was 0·39 for the contribution to energy (Table 4) and 0·44 for the amount consumed (Table 5).

The largest day-to-day variation was found for the following food groups: potatoes; composite dishes; and fish. For these food groups, the ICC were lowest in both recall methods for energy (Supplementary Table S1) and for amount eaten in weight (g) (Supplementary Table S2).

## Discussion

In this study, we evaluated the performance of the self-administered web-based 24 hR method of the first version of the Dutch tool Compl-eat™ against the interviewer-administered telephone-based 24 hR method. We compared the intakes of energy, nutrients and foods, on the assumption that the average of three independent recall days represents usual intake. A lower number of foods were reported in the web-based 24 hR.

Overall, we found lower intakes of about 10 % estimated by the web-based 24 hR compared with the telephone-based 24 hR. The associations determined with LCC between the web-based and the telephone-based 24 hR were reasonably good for alcohol, and fair for energy, most of the macronutrients and some of the micronutrients. The ICC as an estimate of day-to-day variation were similar for the two modes of administration. The lower intakes in the web-based recalls can be explained by a lower number of total reported foods and lower estimates of intake of specific food groups, especially of the food group fats, oils and savoury sauces, followed by sugar and confectionery, cheese, and milk and milk products. From previous research, we know that many participants find it difficult to report the intake of foods from these food groups accurately. Thus, the lack of help from a dietitian may also have contributed to the lower intakes in the web-based 24 hR.

The self-administered web-based 24 hR is a relatively new method, but several tools based on this method have already been evaluated against interviewer-based 24 hR^(^^7^^)^.

Most of the evaluation studies showed better agreement between the intake assessed by the web-based tool and the interviewer-based 24 hR than our study did. However, this may be partly explained by differences in study design, especially the time-frame for scheduling the dietary assessment methods, and also the selected reference method. Thompson *et al*.^(^^25^^)^ found on average a small difference of 2 % in EI between an interview-based 24 hR and their web-based ASA24 method. Per participant, both 24 hR methods were conducted in random order with only 5 to 7 weeks between both recalls. Touvier *et al*. compared one NutriNet-Santé web-based self-administered 24-h record with one 24 hR carried out by a dietitian the next day, both covering the same recall day. The Pearson's correlations for energy were 0·86 for men and 0·85 for women^(^^12^^)^. In Liu *et al*.’s^(^^10^^)^ study, the web-based Oxford web-Q was directly followed by the interview. They found a difference of 0·1 % for energy, and the Spearman's correlation for energy was 0·58. Finally, Zoellner *et al*.^(^^13^^)^ found a correlation of 0·74 for energy between their interactive multimedia dietary assessment tool (IMM) and an interviewer-administered recall. Both the IMM and the interview recall were conducted in random order on the same day. In our study, both the three web-based and the three telephone-based 24 hR were randomly conducted over a long period of time (on average 11 months, range 3–28 months, between the first and the last recall) in order to take seasonal variation into account and evaluate usual intake. This information is important for epidemiological studies. Thus, different types of evaluation studies that provide information about the relative performance of the methods used can be distinguished. One type concerns a short time-frame in which actual dietary intake data are evaluated using two different methods. This type of evaluation is used by Touvier *et al*.^(^^1^,^2^^)^, Liu *et al*.^(^^10^^)^ and Zoellner *et al.*^(^^13^^)^. As these studies did not use independent recall days, or even used the same day for both methods, good agreements between the methods could be expected. Another type of study concerns a long time-frame in which the usual dietary intake is evaluated, assessed by two different methods. This type of evaluation was performed by Thompson *et al*.^(^^25^^)^ and also by our study. However, the number of recalls and the time-frame in Thompson *et al*.’s study were smaller (two recalls in 5 to 7 weeks) than in our study (six recalls in 1 year). Our study may therefore better reflect an evaluation of usual intake.

The evaluation studies described above, including our own study, used a self-report method as the reference method. Another approach is to use a biomarker as reference. This was done by Arab *et al*.^(^^26^^)^, who evaluated their web-based DietDay 24 hR tool against doubly-labelled water (DLW). Six DietDay 24 hR were collected within 2 weeks: three of the 24 hR were collected during a study visit, the other three at home. In this 2-week period, the DLW measurement was also performed. Compared with DLW, the DietDay tool underestimated EI on average by about 10 %, and the Pearson correlation for EI was somewhat smaller than the LCC in our study (0·44 as compared with 0·58 in our study). However, our correlation coefficients may be somewhat inflated because of errors that are similarly present in both the self-administered web-based and the interviewer-administered telephone-based recall method. These correlated errors may have occurred and incorrectly improved the agreement between the methods because both methods depend on the participants’ memory and use the same food composition database and the same database of household measures and standard portion sizes.

Nevertheless, we chose to compare the performance of the web-based recall with that of a telephone-based recall because, in future, we would like to use self-administered web-based 24 hR instead of interviewer-administered telephone-based 24 hR.

Ideally, dietary intakes assessed by our web-based tool should be compared with an objective measure of intake such as a recovery biomarker. Wardenaar *et al*.^(^^27^^)^ evaluated protein intake assessed by Compl-eat™ against N excretion in urine, but in a very specific population of athletes.

Differences between the two 24 hR in our study may be diminished by improving the food list in the web-based tool. The food list consists of the foods in the Dutch food composition table, synonyms, and added foods and recipes derived from participants’ self-reports in previous research. As there are many more foods available on the market than present in our tool, the participants may have had difficulty finding the actual food consumed. Although the web-based recall has the option of adding notes in which the participant can provide information about the actual foods consumed, some participants may not have reported such foods. This hypothesis is supported by the smaller number of reported foods when the web-based tool was used. Including more foods on the food list may help to solve this problem. However, a longer list of foods may confuse users or make it even more difficult to find the correct food – a problem that was also found in the development of the UK online tool myfood24^(^^9^^)^.

Another problem may have been the estimation of portion sizes. In some other web-based tools, pictures or interactive images are used to help the participants to quantify the amount eaten of a food^(^^8^^,^^9^^,^^11^^–^^13^^)^. Compl-eat™ does not contain images; rather, it contains different serving units and portion sizes per food, for instance glasses of different volumes. Also, the descriptions of the serving units may not have been clear enough for the participants. They may have selected for instance the first serving size on the list, whereas a dietitian would have obtained a more accurate estimate by asking additional questions. Moreover, showing all serving sizes of a food at the same time may be better than the pull-down menu used in Compl-eat™^(^^28^^)^.

With respect to response errors, it is questionable whether these errors were similar for the two methods. Errors in reporting the correct type and/or amount of food may be greater in the web-based 24 hR than in the telephone-based 24 hR, as there was no dietitian to help. Respondents may have reported different foods than actually consumed. We found that respondents using the self-administered tool reported fewer foods, so we can hypothesise that errors could occur due to omitted foods and/or beverages. Response errors may also include socially desirable answering, but its influence may be limited as there was no interviewer to please, although some level of social desirability may still exist simply as a consequence of being monitored. Also, under-reporting due to socially desirable answering can be expected more in BMI categories >25 kg/m^2(^^29^^)^. However, we found very small differences in EI when we compared the methods between normal-weight people, overweight people and obese people (7·9, 8·3 and 8·8 %, respectively). To reduce response errors in the tool, we included recall cues at the end of the 24 hR. However, this may not have been sufficient.

Participants with low reading and computer literacy may encounter problems filling in the self-administered 24 hR. Participants with a low or intermediate education level indeed had a larger difference in EI (on average 11·7 %) between the two methods than those with a high education level had (6·6 %).

On the basis of this evaluation, some improvements have already been made to the first version of our tool. It has been made easier to select frequently used foods. For instance, the first time that spread on bread is reported, participants have to enter the type of spread and the amount. At the next consumption time, they can indicate that it is the same type and amount as previously mentioned. This may encourage participants to report every serving consumed. We have also added more recall cues for reporting specific foods, such as fats used for food preparation. In the first version of the tool, recall cues were shown only at the end of the 24 hR; now they are included after each eating occasion in order to better mimic interviewer probes.

Future improvements will include the extension of the food list with more foods, synonyms and recipes. At the same time, the structure of the search lists needs attention in order to maintain easy searching. Finally, we are working on a more simplified version of the tool to increase the user-friendliness for specific populations such as the elderly and people with a low education level. Examples of adaptations to the tool include making it possible to enlarge the font size for better readability, marking the search input (bold font) in the search list, or disabling the recipe module and adding more standard recipes to the food list. Whether all these changes will lead to a better version of Compl-eat™ has to be confirmed in a new validation study, preferably by comparison with recovery markers.

Both participant and researcher burden should be taken into account in the choice of the recall method. Regarding the time aspect, the participant will need about 10 or 15 min more to complete a web-based 24 hR than a telephone-based recall, whereas the researcher will save more than 1 h per recall when using the web-based recall. The first version of Compl-eat™ did not provide information of the time needed for a participant to complete a web-based 24 hR. Ongoing studies using the new version of Compl-eat™ show an average completion time of 40–45 min (range 24–59 min), whereas the telephone-based 24 hR took only 20–30 min for participants. It must be noted, however, that the completion time of the web-based recall captured total login time, and not only the time for filling in the questionnaire and viewing the two instruction videos (4 min 42 s for both videos). However, participants could have skipped the instruction videos, or could have watched them several times. For the researchers, checking the web-based 24 hR and processing the notes took them about 5–10 min per recall, whereas a telephone 24 hR took on average 1·5 h for the interview and coding. Although the time burden is higher for the participants, a web-based recall has the advantage that participants can fill in the recall at a time of their own choosing. Contacting participants for clarifications or to help them complete the recall might improve the quality of the data but will increase the time burden for participants as well as for researchers. In the NQplus study, it was decided not to contact the participants because we think that this is not feasible in large-scale studies.

Thus, in large-scale nutritional epidemiological studies, applying the self-administered 24 hR tool is more feasible than applying an interviewer-administered 24 hR because, with the same staff time, more participants can be included and/or more recalls per participant can be collected. Therefore, the self-administered 24 hR tool allows a better statistical power provided that the validity of the web-based 24 hR is similar to that of a telephone-based 24 hR. Therefore, we conclude that Compl-eat™, a Dutch web-based self-administered 24-h dietary recall, may be a useful tool for large-scale studies after the suggested improvements have been implemented and evaluated.

## Supplementary material

For supplementary material accompanying this paper visit http://dx.doi.org/10.1017/jns.2017.45. click here to view supplementary material

## References

[1] Illner AK, Freisling H, Boeing H, (2012) Review and evaluation of innovative technologies for measuring diet in nutritional epidemiology. Int J Epidemiol 41, 1187–1203. 22933652 10.1093/ije/dys105

[2] Prentice RL, Mossavar-Rahmani Y, Huang Y, (2011) Evaluation and comparison of food records, recalls, and frequencies for energy and protein assessment by using recovery biomarkers. Am J Epidemiol 174, 591–603. 21765003 10.1093/aje/kwr140 PMC3202154

[3] Subar AF, Kipnis V, Troiano RP, (2003) Using intake biomarkers to evaluate the extent of dietary misreporting in a large sample of adults: the OPEN study. Am J Epidemiol 158, 1–13. 12835280 10.1093/aje/kwg092

[4] Kipnis V, Subar AF, Midthune D, (2003) Structure of dietary measurement error: results of the OPEN biomarker study. Am J Epidemiol 158, 14–21; discussion 22–16. 12835281 10.1093/aje/kwg091

[5] Willet W (2013) Nutritional Epidemiology, 3rd ed., vol. 40, pp. 49–69. Oxford: Oxford University Press.

[6] Zimmerman TP, Hull SG, McNutt S, (2009) Challenges in converting an interviewer-administered food probe database to self-administration in the National Cancer Institute Automated Self-administered 24-hour recall (ASA24). J Food Compost Anal 22, Suppl. 1, S48–S51. 20161418 10.1016/j.jfca.2009.02.003 PMC2786178

[7] Timon CM, van den Barg R, Blain RJ, (2016) A review of the design and validation of web- and computer-based 24-h dietary recall tools. Nutr Res Rev 29, 268–280. 27955721 10.1017/S0954422416000172

[8] Arab L, Wesseling-Perry K, Jardack P, (2010) Eight self-administered 24-hour dietary recalls using the Internet are feasible in African Americans and Whites: the energetics study. J Am Diet Assoc 110, 857–864. 20497774 10.1016/j.jada.2010.03.024 PMC2909478

[9] Carter MC, Albar SA, Morris MA, (2015) Development of a UK online 24-h dietary assessment tool: myfood24. Nutrients 7, 4016–4032. 26024292 10.3390/nu7064016 PMC4488770

[10] Liu B, Young H, Crowe FL, (2011) Development and evaluation of the Oxford WebQ, a low-cost, web-based method for assessment of previous 24 h dietary intakes in large-scale prospective studies. Public Health Nutr 14, 1998–2005. 21729481 10.1017/S1368980011000942

[11] Subar AF, Kirkpatrick SI, Mittl B, (2012) The Automated Self-Administered 24-hour dietary recall (ASA24): a resource for researchers, clinicians, and educators from the National Cancer Institute. J Acad Nutr Diet 112, 1134–1137. 22704899 10.1016/j.jand.2012.04.016 PMC3721511

[12] Touvier M, Kesse-Guyot E, Mejean C, (2011) Comparison between an interactive web-based self-administered 24 h dietary record and an interview by a dietitian for large-scale epidemiological studies. Br J Nutr 105, 1055–1064. 21080983 10.1017/S0007114510004617

[13] Zoellner J, Anderson J & Gould SM (2005) Comparative validation of a bilingual interactive multimedia dietary assessment tool. J Am Diet Assoc 105, 1206–1214. 16182635 10.1016/j.jada.2005.05.011

[14] Conway JM, Ingwersen LA, Vinyard BT, (2003) Effectiveness of the US Department of Agriculture 5-step multiple-pass method in assessing food intake in obese and nonobese women. Am J Clin Nutr 77, 1171–1178. 12716668 10.1093/ajcn/77.5.1171

[15] Sluik D, Brouwer-Brolsma EM, de Vries JHM, (2016) Associations of alcoholic beverage preference with cardiometabolic and lifestyle factors: the NQplus study. BMJ Open 6, e010437. 10.1136/bmjopen-2015-010437 PMC4916604 27311903

[16] Donders-Engelen MVdHL & Hulshof K (2003) Maten, Gewichten en Codenummers 2003. Food Portion Sizes and Coding Instructions. Zeist: Wageningen University and TNO Nutrition.

[17] van Rossum CTM, Fransen HP, Verkaik-Kloosterman J, (2011) *Dutch National Food Consumption Survey 2007–2010. Diet of Children and Adults Aged 7 to 69 Years. RIVM-Rapport 350050006*. Bilthoven: RIVM.

[18] NEVO-tabel (2011) Dutch Food Composition Database. Den Haag: RIVM/Voedingscentrum.

[19] Willett WC, Howe GR & Kushi LH (1997) Adjustment for total energy intake in epidemiologic studies. Am J Clin Nutr 65, Suppl. 4, S1220–S1228. 10.1093/ajcn/65.4.1220S 9094926

[20] Bland JM & Altman DG (1986) Statistical methods for assessing agreement between two methods of clinical measurement. Lancet i, 307–310. 2868172

[21] Lin LI (1989) A concordance correlation coefficient to evaluate reproducibility. Biometrics 45, 255–268. 2720055

[22] Monin B & Oppenheimer DM (2005) Correlated averages vs. averaged correlations: demonstrating the warm glow heuristic beyond aggregation. Soc Cognit 23, 257–278.

[23] Black AE (2000) Critical evaluation of energy intake using the Goldberg cut-off for energy intake: basal metabolic rate. A practical guide to its calculation, use and limitations. Int J Obes Relat Metab Disord 24, 1119–1130. 11033980 10.1038/sj.ijo.0801376

[24] Henry CJ (2005) Basal metabolic rate studies in humans: measurement and development of new equations. Public Health Nutr 8, 1133–1152. 16277825 10.1079/phn2005801

[25] Thompson FE, Dixit-Joshi S, Potischman N, (2015) Comparison of interviewer-administered and automated self-administered 24-hour dietary recalls in 3 diverse integrated health systems. Am J Epidemiol 181, 970–978. 25964261 10.1093/aje/kwu467 PMC4462333

[26] Arab L, Tseng CH, Ang A, (2011) Validity of a multipass, web-based, 24-hour self-administered recall for assessment of total energy intake in blacks and whites. Am J Epidemiol 174, 1256–1265. 22021561 10.1093/aje/kwr224 PMC3224251

[27] Wardenaar FC, Steennis J, Ceelen IJ, (2015) Validation of web-based, multiple 24-h recalls combined with nutritional supplement intake questionnaires against nitrogen excretions to determine protein intake in Dutch elite athletes. Br J Nutr 114, 2083–2092. 26435534 10.1017/S0007114515003839

[28] Subar AF, Crafts J, Zimmerman TP, (2010) Assessment of the accuracy of portion size reports using computer-based food photographs aids in the development of an automated self-administered 24-hour recall. J Am Diet Assoc 110, 55–64. 20102828 10.1016/j.jada.2009.10.007 PMC3773715

[29] Trijsburg L, Geelen A, Hollman PC, (2017) BMI was found to be a consistent determinant related to misreporting of energy, protein and potassium intake using self-report and duplicate portion methods. Public Health Nutr 20, 598–607. 27724995 10.1017/S1368980016002743 PMC10261408

