# Supplementary material for: Evaluation of dietary intake assessed by the Dutch self-administered web-based dietary 24-h recall tool (Compl-eat™) against interviewer-administered telephone-based 24-h recalls
Source: J Nutr Sci. 2017 Sep 19;6:e49. doi: 10.1017/jns.2017.45 (PMC5672320; doi:10.1017/jns.2017.45)
Supplement: Supplementary file 1 [file S2048679017000453sup.zip › S2048679017000453sup001.pdf]

Supplementary Table 2. Intra-class correlation coefficients in amount eaten (grams) of the different food groups between the three self-administered web-based and the three interviewer-administered telephone-based 24-hour recalls (n=514)

Amount eaten (grams/day)	Web-based 24-hour recalls			Telephone-based 24-hour recalls		
		95% Confidence interval			95% Confidence interval	
	ICC	Lower bound	Upper bound	ICC	Lower bound	Upper bound
Alcoholic beverages	0.51	0.46	0.56	0.46	0.41	0.51
Bread	0.45	0.39	0.50	0.46	0.41	0.51
Cereal products and binding agents	0.10	0.04	0.15	0.12	0.06	0.17
Cheese	0.23	0.17	0.29	0.23	0.17	0.29
Coffee, tea, and water	0.62	0.58	0.66	0.54	0.49	0.59
Composite dishes	0.04	-0.01	0.09	0.02	-0.03	0.08
Eggs	0.16	0.10	0.21	0.16	0.10	0.22
Fats, oils, and savoury sauces	0.19	0.13	0.24	0.08	0.02	0.13
Fish	0.07	0.02	0.13	0.11	0.05	0.16
Fruit	0.39	0.34	0.45	0.38	0.33	0.44
Fruit/vegetable juices, soft drinks	0.43	0.37	0.48	0.45	0.39	0.50
Legumes	0.10	0.05	0.16	0.08	0.03	0.14
Meat, meat products, and poultry	0.20	0.15	0.26	0.19	0.14	0.25
Milk and milk products	0.49	0.44	0.54	0.48	0.43	0.53
Nuts, seeds and snacks	0.14	0.09	0.20	0.15	0.09	0.20
Pastry, cake, and biscuits	0.18	0.13	0.24	0.20	0.14	0.26
Potatoes	0.08	0.03	0.13	0.09	0.04	0.14
Savoury sandwich fillings	0.32	0.26	0.37	0.37	0.32	0.43
Soups	0.14	0.09	0.20	0.14	0.09	0.20
Soya and vegetarian products	0.57	0.53	0.62	0.63	0.58	0.67
Sugar and confectionary	0.29	0.24	0.35	0.33	0.27	0.38
Vegetables	0.17	0.12	0.23	0.27	0.22	0.33

ICC, Intra-class correlation coefficient.
